# Relation of Prenatal Air Pollutant and Nutritional Exposures with Biomarkers of Allergic Disease in Adolescence

**DOI:** 10.1038/s41598-018-28216-0

**Published:** 2018-07-12

**Authors:** Joanne E. Sordillo, Karen M. Switkowski, Brent A. Coull, Joel Schwartz, Itai Kloog, Heike Gibson, Augusto A. Litonjua, Jennifer Bobb, Petros Koutrakis, Sheryl L. Rifas-Shiman, Emily Oken, Diane R. Gold

**Affiliations:** 1 Division of Chronic Disease Research Across the Lifecourse (CoRAL), Department of Population Medicine, Harvard Medical School and Harvard Pilgrim Health Care Institute, Boston, MA USA; 2 Department of Biostatistics, Harvard T.H. Chan School of Public Health, Boston, Massachusetts USA; 3 Department of Environmental Health, Harvard T.H. Chan School of Public Health, Boston, Massachusetts USA; 4 Division of Pediatric Pulmonary Medicine, University of Rochester Medical Center, Rochester, NY USA; 5 Kaiser Permanente Washington Health Research Institute, Seattle, WA USA; 6 Channing Division of Network Medicine, Brigham and Women’s Hospital, Harvard Medical School, Boston, Massachusetts USA

## Abstract

Prenatal exposures may be critical for immune system development, with consequences for allergic disease susceptibility. We examined associations of prenatal exposures (nutrient intakes and air pollutants) with allergic disease biomarkers in adolescence. We used data from 857 mother-child pairs in Project Viva, a Massachusetts-based pre-birth cohort. Outcomes of interest at follow-up (median age 12.9 years) were fractional exhaled nitric oxide (FeNO) and total serum IgE. We applied Bayesian Kernel Machine Regression analyses to estimate multivariate exposure-response functions, allowing for exposure interactions. Exposures were expressed as z-scores of log-transformed data and we report effects in % change in FeNO or IgE z-score per increase in exposure from the 25^th^ to 75^th^ percentile. FeNO levels were lower with higher intakes of prenatal vitamin D (−16.15%, 95% CI: −20.38 to −2.88%), folate from foods (−3.86%, 95% CI: −8.33 to 0.83%) and n-3 PUFAs (−9.21%, 95% CI −16.81 to −0.92%). Prenatal air pollutants were associated with higher FeNO and IgE, with the strongest associations detected for PM_2.5_ with IgE (25.6% increase, 95% CI 9.34% to 44.29%). We identified a potential synergistic interaction (p = 0.02) between vitamin E (food + supplements) and PM_2.5_; this exposure combination was associated with further increases in FeNO levels.

## Introduction

Allergic diseases represent a large proportion of childhood chronic disease, and constitute a major public health burden. Susceptibility to childhood allergic disease likely begins in fetal life. Fetal immune cell precursors are detectable at 3–4 weeks gestation, and begin seeding fetal tissues soon after^1^. Myeloid and lymphoid cell populations continue to expand until birth, and allergic sensitization and airway inflammation in response to foreign antigen exposures evolves throughout early and later childhood, further widening the potential window of susceptibility^2^.

Exposures encountered during prenatal life have been shown to have either adverse or, in some cases, protective associations with asthma and allergic disease in later childhood^3,4^. For example, a number of studies have shown early life air pollution exposures to be risk factors for later wheeze, asthma, and reduced lung function^5–7^. Other studies have found protective associations of maternal antenatal vitamin D or antioxidant nutrient intake with later allergic or respiratory outcomes^8–12^, though their findings have not always been reproducible^13–16^. In the antenatal and early-life periods, exposures do not occur one at a time, but as mixtures, with possible interactions amongst the exposures within those mixtures. Between-study variation in associations of individual exposures with allergy, asthma symptoms and lung function may occur in part because their effects in the context of other exposures were not explored. In addition, between-study variation in associations of exposures with allergy or asthma outcomes may reflect variation in subject sensitivity to those exposures and their mixtures. Recent studies have suggested sexual dimorphism in allergy and asthma that may lead to differential responses to pollutants^17^.

IgE and fractional exhaled nitric oxide (FeNO) are important intermediate biomarkers of allergic disease and allergic airway inflammation. IgE mediates the type I hypersensitivity reactions involved in pathogenesis of asthma and allergic rhinitis^18^. FeNO is a known biomarker of eosinophilic airway inflammation, and is elevated in subjects with atopy^19^. In adolescent children followed since the prenatal period, we examined multiple components from two prenatal exposure types, air pollution and maternal nutrient intakes, to determine their associations with IgE and FeNO measured in adolescence. We hypothesized that intake of prenatal antioxidant nutrients would mitigate the detrimental effects of prenatal air pollutants on allergic disease biomarkers measured in adolescence. We considered nutrient intakes from diet alone (as bioavailability and potential protective effects may be greatest when nutrients are absorbed from food), and then also conducted analyses using combined nutrient intakes from foods plus supplements. We also examined the potential for sexual dimorphism in the effects of mixtures of exposures on outcomes in adolescence, when the influences of sex on allergy and allergic airway inflammatory responses may be in transition.

To investigate these hypotheses, we applied Bayesian Kernel Machine Regression (BKMR)^20^, a recently developed approach for estimating the health effects of multi-exposure mixtures. Although there are other types of exposure-mixture modeling approaches, many of these have significant limitations. For example, clustering methods are often too reductionist (categorizing continuous exposures), variable selection methods (i.e. Lasso regression) typically rely on simple linear and additive models that may not adequately capture complex exposure-response relationships, and traditional hierarchical modeling techniques, while accounting for correlation amongst exposure mixture components, are typically also based on a parametric model that assumes (often unrealistic) linear and additive associations. BKMR offers distinct advantages over these other approaches, including the ability to allow for potential non-linear exposure-response relationships, to identify interactions between exposures, and to simultaneously rank the importance of hierarchical exposure groups in association with the outcome of interest. We applied BKMR methodology to data from Project Viva, a longitudinal pre-birth cohort study of prenatal exposures and chronic disease outcomes, with follow-up through adolescence.

## Results

Sociodemographic and perinatal characteristics of the mothers and children enrolled in the study are shown in Table 1. Mean maternal pre-pregnancy BMI was 24.7 kg/m^2^. Sixty six percent of the children in the study were white, 15% were black, 4% were Hispanic and 16% were other race/ethnicities. Nine percent of women reported smoking in pregnancy. Characteristics of the mother-infant pairs at enrollment were very similar to the characteristics of those with FeNO and total IgE outcome data at the early teen visit age 12 (Supplemental Table 1). Distributions of the prenatal nutrient and air pollutant exposures (25^th^ percentile, median, 75^th^ percentile) are shown in Table 2.

**Table 1:** FeNO and Total Serum IgE levels in Adolescence by Participant Characteristics.

Characteristic	Subjects N (%), For subjects with FeNO	Geometric Mean FeNO (ppb) at age 11.9 to 16.6 N = 857 subjects	Geometric Mean Total Serum IgE (IU/ml) at age 11.9–16.6 N = 590 subjects
**Smoking in Pregnancy**			
Never	598 (69%)	18.5	62.0
Smoked during Pregnancy	78 (9%)	22.1	89.8
Former	181 (21%)	19.0	56.7
**Maternal Hay fever**			
Yes	256 (30%)	19.7	79.9
No	600 (70%)	18.5	56.9*
**Child’s Race/Ethnicity**			
Black	123 (15%)	19.8	83.5
White	565 (66%)	18.2	56.9
Hispanic	35 (4%)	19.5	53.6
Other	134 (16%)	21.3	76.4
**Child’s sex**			
Female	429 (50%)	17.9	58.1
Male	428 (50%)	20.0*	68.2
**Season of Birth**			
Winter	203 (24%)	20.6	65.5
Spring	218 (25%)	19.6	66.5
Summer	249 (29%)	17.7	67.6
Fall	187 (22%)	17.9	51.8

*p value < 0.05 for t-test/ANOVA.

**Table 2:** Distribution of Prenatal Exposure Variables (Nutrient Intakes from Foods and Supplements and Air Pollutant Exposures).

Prenatal Exposure Variable	N	25^th^ Percentile	Median	75^th^ Percentile
**Vitamin C**, mg/d (Foods only)	857	132.6	168.7	212.5
mg/d (Food + Supplements)	857	206.9	254.1	308.9
**Vitamin E**, mg/d (Foods only)	857	5.4	6.4	8.0
**Vitamin E**, mg/d (Foods + Supplements)	857	13.6	18.3	25.1
**Beta Carotene**, μg/d (Foods only)	857	2432.4	3521.8	4934.9
**Beta Carotene**, μg/d (Foods + Supplements)	857	3251.3	4402.6	5875.5
**Folate**, μg/d (Foods only)	857	287.4	351.3	421.1
**Folate**, μg/d (Foods + Supplements)	857	874.2	1125.1	1311.2
**Choline** mg/d (Foods only)	857	290.3	323.1	361.0
**Vitamin D**, IU/d (Foods only)	857	150.1	208.6	280.1
**Vitamin D**, IU/d (Foods + Supplements)	857	432.8	552.1	657.7
**n- 6 PUFAs**, gm/day (Foods only)	857	10.0	11.7	13.6
**n-3 PUFAs** gm/day (Foods only)	857	0.9	1.1	1.3
**Black Carbon** μg/m^3^	857	0.5	0.7	0.8
**PM**_2.5_ μg/m^3^	766	10.6	11.7	12.9

Geometric mean (+/− stddev) FeNO concentrations were 19 ppb (+/− 2 ppb), and IgE was 63.2 IU/ml (+/− 4.14). Associations between socioeconomic and demographic covariates and our outcomes of interest are shown in Table 1. Males had higher FeNO as compared to females, but sex was not a predictor of total serum IgE levels. Children of other race/ethnicity tended to have higher FeNO and IgE, and there was a suggestion of an effect for higher IgE in black children. Differences between racial groups overall were marginally statistically significant (ANOVA p values = 0.1 for group comparisons). Participants born in winter or spring tended to have higher FeNO, but season of birth was not associated with total IgE levels. Maternal history of hay fever was related to a higher offspring total serum IgE concentration in adolescence (p = 0.006). There was no evidence for associations between report of smoking in pregnancy or maternal BMI and either FeNO or total serum IgE in adolescence.

For BKMR analyses, posterior inclusion probabilities were calculated for exposure groups in models for each outcome. In models for FeNO, the largest group of nutrients (Vitamin D, Vitamin C, Folate, n−3 and n−6 polyunsaturated fatty acids (PUFAs)) was consistently ranked higher than air pollutant exposures, with the second nutrient grouping (Vitamin E and β-carotene) ranked last. (Supplemental Table 2) In models for IgE, air pollutant exposures had much higher posterior inclusion probabilities than either of the individual nutrient groups, which showed similar posterior inclusion probabilities to one another. (Supplemental Table 3).

**Figure 1:**
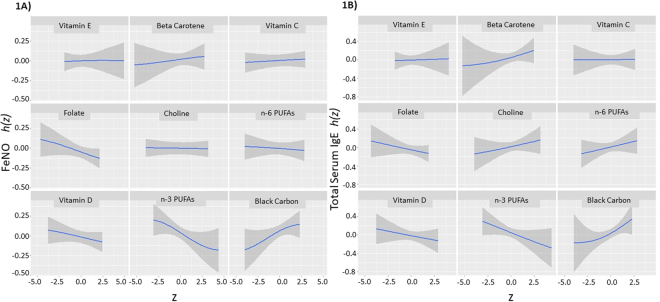
Estimated exposure response functions (*h*(z)) for prenatal nutrient intake (foods only), prenatal air pollution (Black carbon) and allergic disease outcomes in adolescence. Plots show the estimated relationship between log-transformed z scores of exposures (z) and outcomes; shaded areas indicate 95% credible intervals. Plots are shown for outcomes FeNO (**A**) and Total Serum IgE (**B**).

For models of FeNO, estimated dose response curves for individual exposures are shown in Fig. 1, and point estimates for percent increase in FeNO z score, with an increase in exposure from the 25^th^ to 75^th^ percentile, (with all other exposures are fixed at the median) are shown in Table 3. Maternal prenatal n−3 polyunsaturated fatty acids and folate from food sources were associated with reduced early teen FeNO levels (−9.21% (95% CI −16.81% to −0.92%) for n-3s and −3.86%, (95% CI −8.33% to 0.83%) for folate. In models with IgE as the outcome, point estimates for these two nutrients and were similar in magnitude and direction (Table 3). Prenatal vitamin D intake from foods showed a trend toward decreased FeNO measures in adolescence (Table 3). Overall, the strongest association observed was for black carbon (BC) exposure and total IgE level (15.25% increase in total IgE was associated with an interquartile range increase in log BC exposure). In models for the FeNO outcome, the point estimate for BC was 7.39% (95% CI −1.35 to 16.91%).

**Table 3:** BKMR Estimates for associations of Prenatal Nutrient Intakes (Foods Only) and Prenatal Air Pollutant (3^rd^ Trimester Black Carbon) Exposure with Allergic Disease Outcomes (FeNO and Total Serum IgE) in adolescence.

Hierarchical Exposure Group	Prenatal Exposures	Health Outcomes			
	Nutrient Intakes from Foods Only and Air Pollution	FeNO* (% change per interquartile range increase in exposure)		Total Serum IgE* (% change per interquartile range increase in exposure)	
		*Est*.	*95% CI*	*Est*.	*95% CI*
1	Vitamin E	0.33%	−3.90 to 4.75%	0.56%	−7.04 to 8.77%
	Beta-carotene	1.91%	−4.81 to 9.11%	6.83%	−4.63 to 19.66%
2	Vitamin C	0.74%	−2.45 to 4.03%	0.33%	−8.52 to 10.04%
	Folate	−3.86%	−8.33% to 0.83%	−4.34%	−12.12 to 4.13%
	Choline	−0.23%	−2.83 to 2.43%	5.34%	−4.98 to 16.77%
	n-6 PUFAs	−1.00%	−5.84 to 4.08%	6.09%	−5.41 to 18.99%
	Vitamin D	−3.24%	−8.39 to 2.19%	−4.88%	−14.55 to 5.89%
	n-3 PUFAs	**−9.21%**	**−16.81 to −0.92%**	−10.51%	−21.26 to 1.69%
3	Black Carbon	7.39%	−1.35 to 16.91%	**15.25%**	**1.92 to 30.33%**

*Model adjusted for maternal pre-pregnancy BMI, education, hay fever, and pregnancy smoking status (never, former, smoked during pregnancy); season of birth, sine and cosine of date at outcome measurement; and child’s race/ethnicity and sex. Estimates with p  < 0.05 are in bold.

**Figure 2:**
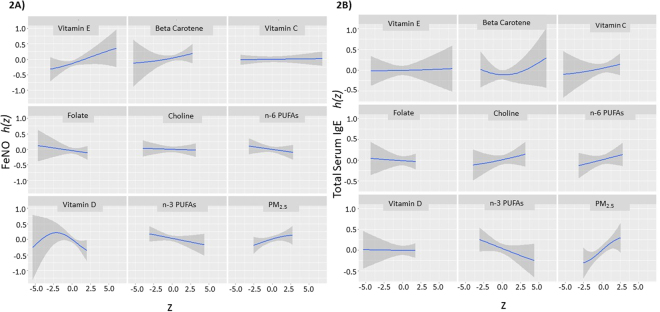
Estimated exposure response functions (*h*(z)) for prenatal nutrient intake from foods alone (choline, n-6 PUFAs, n-3 PUFAs, Beta-carotene) and foods + supplements (Vitamin C, Vitamin E, Vitamin D, Folate), prenatal air pollution (PM_2.5_) and allergic disease outcomes in adolescence. Plots show the estimated relationship between log-transformed z scores of exposures (z) and outcomes; shaded areas indicate 95% credible intervals. Plots are shown for outcomes FeNO (**A**) and Total Serum IgE (**B**).

**Figure 3:**
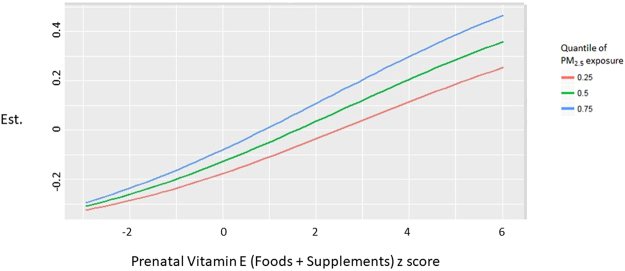
Prenatal Vitamin E Intake (Foods + Supplements) may interact with PM_2.5_ Exposure to increase FeNO levels in adolescence. In subjects with elevated prenatal exposure to PM_2.5_, prenatal Vitamin E shows a stronger association with FENO in adolescence.

We also considered nutrient exposures as combined intakes from food and supplements, if supplement intakes for a given nutrient were available in our data (Table 4). Vitamin D intake (food plus supplements) showed a strong inverse association with FeNO (−16.15%, 95% CI: −20.38 to −2.88%). The shape of the estimated dose-response curve was not linear (Fig. 2), with a relatively flat dose-response at lower vitamin D levels, and a decreasing linear slope from moderate to high Vitamin D levels. In contrast, there was no clear relationship between Vitamin D intake from foods plus supplements and total IgE level (Table 4, Fig. 3). The point estimate for prenatal vitamin E intake from food and supplements combined was 6.94% (95% CI −2.36 to 12.24%) for the FeNO outcome (Table 4), which was much larger than the vitamin E estimate from diet alone (Table 3). No association was observed for Vitamin E and total IgE levels.

**Table 4:** BKMR Estimates for associations of Prenatal Nutrient Intakes (Foods + Supplements) and Prenatal Air Pollutant (3^rd^ Trimester PM2.5) Exposure with Allergic Disease Outcomes (FeNO and Total Serum IgE) in adolescence.

Hierarchical Exposure Group	Prenatal exposures	Health Outcomes			
	Nutrients*, Air Pollutants	FeNO** (% change per interquartile range increase in exposure)		Total Serum IgE** (% change per interquartile range increase in exposure)	
		Est.	*95% CI*	*Est*.	*95% CI*
1	**Vitamin E** (Foods + Supplements)	6.94%	−2.36 to 12.24%	−1.01%	−11.40% to 10.60%
	**Beta-carotene** (Foods)	5.86%	−3.54 to 12.65%	4.50%	−6.44% to 16.73%
2	**Vitamin C** (Foods + Supplements)	0.33%	−2.06 to 2.59%	0.38%	−7.33% to 8.72%
	**Folate** (Foods + Supplements)	−3.30%	−9.30 to 4.98%	−1.11%	−9.81% to 8.42%
	**Choline** (Foods)	−0.91%	−5.68 to 4.61%	4.62%	−5.33% to 15.61%
	**n−6 PUFAs** (Foods)	−4.17%	−9.77 to 4.15%	5.79%	−5.48% to 18.40%
	**Vitamin D** (Foods + Supplements)	**−16.15%**	**−20.38 to −2.88%**	−0.23%	−8.53% to 8.83%
	**n−3 PUFAs** (Foods)	−6.36%	−11.77 to 2.98%	−9.50%	−20.15% to 2.57%
3	**PM**_**2.5**_	10.51%	−0.78% to 16.63%	**25.60%**	**9.34% to 44.29%**

*Supplement data was not available for all nutrients. **Adjusted for maternal pre-pregnancy BMI, education, hay fever, and pregnancy smoking status (never, former, smoked during pregnancy); season of birth, sine and cosine of date at outcome measurement; and child’s race/ethnicity and sex. Estimates with p < 0.05 are in bold.

In addition to modeling exposure to BC as an air pollutant, we also considered exposure to PM_2.5_ (Table 4, Fig. 2). PM_2.5_ was associated with a 10.51% increase in FeNO (95% CI −0.78 to 16.63%) and a 25.6% increase in serum total IgE (95% CI 9.34 to 44.29%). Plots of BKMR results for the effects of one exposure at various levels of another exposure showed an interaction between PM_2.5_ and Vitamin E intake from foods plus supplements. As shown in Fig. 3, Vitamin E is associated with larger increases in FeNO when PM_2.5_ exposure is higher. This interaction was confirmed in a standard linear regression model (p = 0.02 for interaction term). For participants in the highest quartile of PM_2.5_ exposure, Vitamin E (food plus supplements) was associated with 8.42% increase in FeNO (95% CI −1.18 to 13.87%). For participants in the highest quartile of Vitamin E (food plus supplements) exposure, PM_2.5_ was associated with a 14.29% increase in FeNO levels (95% CI 0.49 to 20.94%). In contrast, no interaction between black carbon and vitamin E was observed in BKMR models for the FeNO outcome, and a test of the interaction term in linear regression models was not statistically significant (p = 0.75).

**Figure 4:**
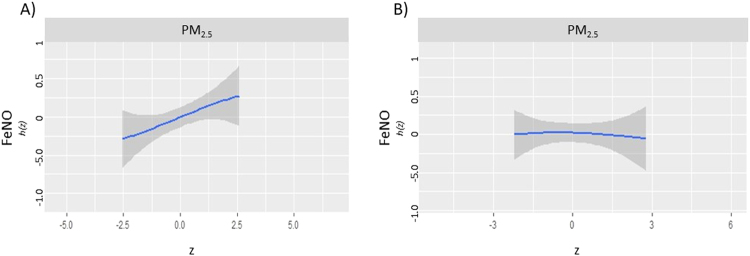
Estimated response function (*h*(z)) for prenatal exposure to PM_2.5_ and FeNO in adolescence vary by sex. Prenatal PM_2.5_ exposure (z) was associated with increased FeNO in males (**A**), but not females (**B**).

In addition to examining interactions amongst the exposures themselves, we examined potential sex interactions by conducting sex-stratified BKMR analyses and comparing sex-specific prenatal exposure-response curves (Fig. 4). Air pollutant exposure response associations were strongest for male participants. In stratified analyses, prenatal PM_2.5_ exposure was associated with FeNO in males (20.47% increase, 95% CI 2.52 to 41.56%) but not in females (−1.52% decrease, 95% CI −15.50 to 14.79%).The interaction between sex and PM_2.5_ exposure was reproduced in a linear model (p < 0.05 for interaction term). We did not detect any differences in exposure response curves by sex for the total serum IgE outcome.

We also examined other exposure combinations in two additional sets of models (models for PM_2.5_ exposure and food-based nutrient intakes (Supplemental Table 4) and models for black carbon exposure and Nutrients (Food + supplements) (Supplemental Table 5)). For brevity, and since main effects of exposures showed similar trends across all models, we present only the models in Table 3 (food-based nutrients and black carbon exposure) and Table 4 (nutrients from food plus supplements and PM_2.5_ exposure) in the main text.

## Discussion

In this work, we used a state of the art, non-parametric statistical methodology, BKMR, to identify potential relationships between exposures mixtures encountered in prenatal life and biomarkers of allergic disease in adolescence. While interactions between prenatal nutrient and air pollutant exposures have been studied in the context of birth defects and childhood cognitive outcomes^21,22^, this is the first study, to our knowledge, that considers how these types of prenatal exposure mixtures may relate to childhood allergic disease. We hypothesized that exposure to prenatal nutrients, particularly antioxidants, would have protective effects, and that these protective nutrients would interact with prenatal air pollutant exposures to diminish their adverse effects on childhood allergic disease. In keeping with our hypothesis, we observed that higher prenatal intakes of n-3 polyunsaturated fatty acids (a potential immunomodulatory agent), vitamin D (an antioxidant and immunomodulatory agent) and folate were associated with lower levels of allergic disease biomarkers. In contrast with other nutrients, total vitamin E intake in pregnancy appeared to amplify the adverse effect of prenatal exposure to PM_2.5_ on FeNO measured in adolescence, suggesting a synergy between these two exposures. The effects of protective nutrients were consistent across all levels of background air pollution exposure, which suggests that there was no statistical interaction between these two exposure types. However, when considering additive effects, it is clear that protective nutrients may help reduce the overall impact of adverse exposures.

In our study, average folate intake over the 1^st^ and 2^nd^ trimesters (specifically from foods) showed a borderline association with lower concentrations of FeNO in adolescence, but showed no association with total serum IgE levels. Prenatal folate intake and allergic disease risk has been examined in multiple previous reports, with inconsistent results. Some reports show no association with folate^23,24^, but others demonstrate associations between prenatal folate and increased risk of atopy, early childhood asthma or eczema^25–29^. Differences across studies may stem from variation in prenatal exposure window (early vs. late pregnancy), underlying genetic susceptibility (maternal history of allergic disease and/or MTHFR genotype), and dose of folate. We observed a potential protective association for higher dietary prenatal folate and lower adolescent FeNO within a fairly low exposure range (150 to 1200 μg/day), which is well below the levels of supplemental folate that have been linked to increased allergic disease risk. Although the potential mechanism of action for folate on asthma/allergy phenotypes has yet to be elucidated, its role as a methyl donor suggests that effects of prenatal folate could be mediated via epigenetic modifications. However, our observation of suggestive protective associations with intake from diet alone, suggests that folate (found naturally in green leafy vegetables and other food), but not folic acid (the form found in supplements), may be responsible for the observed protective associations.

Two other prenatal nutrients with immunomodulatory potential, n-3 polyunsaturated fatty acids and vitamin D, were also associated with lower FeNO levels in our study. n-3 polyunsaturated fatty acids also showed protective effects with similar (although not statistically significant) effect sizes for total serum IgE. In Project Viva, we have previously reported a lower risk of mid childhood atopy and allergic rhinitis with more vitamin D intake in pregnancy^30^. The results of the current study extend these findings by examining a biomarker of allergic disease (FeNO) at a later time point. In addition to our Project Viva findings, results from clinical trials also show reduced childhood allergic disease risk with higher intakes of these prenatal nutrients. A recent randomized controlled trial published in the New England Journal of Medicine showed lower rates of persistent wheeze or asthma in children whose mothers were supplemented with 2.4 g n−3 polyunsaturated fatty acids (in fish oil) vs. olive oil starting at 24 weeks gestation^31^. Secondary analyses of the VDAART randomized trial demonstrate lower odds of early childhood asthma (OR = 0.42, 95% CI 0.19 to 0.91) when prenatal supplementation of 4400 IU/day Vitamin D is administered to women with baseline Vitamin D serum levels of 30 ng/ml or above^32^. Mechanistic evidence exists for how both n-3 polyunsaturated fatty acids and Vitamin D may alter immune phenotypes in utero that complements epidemiological findings. n−3 supplementation in pregnancy has been linked to increased cord blood DNA methylation of genes encoding Th1 and Th2 cytokine production^33^. Vitamin D inhibits Th1-cytokine synthesis and suppresses allergen-specific IgE in *in vitro* studies^34^, and is associated with expression of innate immune receptors (TLR9 and TLR2) in cord blood^35^. Vitamin D may also increase production of IL-10 in neonatal bronchial epithelium^36^. Thus, our findings for nutrients with immunomodulatory potential (Vitamin D and n−3 polyunsaturated fatty acids) and their influence on allergic disease biomarkers in adolescence are supported mechanistically by experimental data, and demonstrate protective associations that extend beyond those observed in clinical trials (which are focused upon early childhood allergic disease outcomes).

In contrast to the other nutrients studied, total prenatal intake of Vitamin E (foods plus supplements) appeared to be associated with higher FeNO in adolescence, particularly in the context of higher PM_2.5_ levels. Prenatal exposure to PM_2.5_ has been associated with greater risk of childhood asthma and reduced lung function in a number of studies^37^; mechanisms of action are hypothesized to include oxidative stress and enhancement of pro-inflammatory cytokine production^37,38^. At high doses, Vitamin E is known to have pro-oxidant effects, which may be induced by the production of α-tocopheroxyl radicals. The pro-oxidant influences of high levels of Vitamin E and PM_2.5_ combined might explain the potential synergy between the two exposures detected in our analysis of FeNO. Interestingly, we did not observe a similar interaction for the total serum IgE outcome in adolescence. Although there is significant overlap between both of the allergic disease biomarkers considered in our study, we may have had more power to detect an association with FeNO. FeNO is more representative of lung-specific Th2 inflammation, while circulating IgE levels are instead a systemic, rather than compartment specific, biomarker. Even though there was no interaction with Vitamin E, PM_2.5_ exposure was as an independent predictor of relatively large increases (~25%) in total serum IgE measured in adolescence.

We also uncovered an interaction with sex. In male participants, we observed a strong association between prenatal PM_2.5_ levels and increased FeNO in adolescence, but exposure response curves for this association were essentially flat in female participants (Fig. 4). Others have reported important sex interactions for PM_2.5_ exposure. Hsu and colleagues showed that prenatal PM_2.5_ exposure is related to increased asthma incidence in males, but not females^17^. Underlying sex differences in prenatal lung development may in part explain the unique susceptibility of males to PM_2.5._

In this work, we chose to focus on 3^rd^ trimester air pollutants because the third trimester is an active period of lung development, with potential import for airway inflammatory outcomes in childhood and adolescence, and because exposure assessments for this trimester were most complete for PM_2.5_. We also tested 1^st^ and 2^nd^ trimester black carbon exposures in our models for FeNO and IgE (data not shown), but found the strongest associations for 3^rd^ trimester exposures.

There were some limitations to our study. For maternal dietary assessment, we used intakes based on food frequency questionnaires from the 1^st^ and 2^nd^ trimester, rather than direct serum measurements. However, the food frequency questionnaire used to capture nutrient intakes has been validated for use in pregnancy, and the assessment of nutrient intakes at two time points strengthens the reliability of our exposure assessments. Prenatal exposure to 3^rd^ trimester air pollutants did not directly overlap with our nutrient assessments from the 1^st^ and 2^nd^ trimesters. It is possible that we would have observed more interaction effects if we had data on 3^rd^ trimester nutrient intakes that were concurrent with air pollutant exposures; however the 1^st^ and 2^nd^ trimester nutrient intakes are likely to be reasonable approximations of dietary intakes in the 3^rd^ trimester. We assessed air pollution exposure by PM_2.5_, and black carbon (a component of PM_2.5_), but did not interrogate additional PM components, many of which may have import for health effects. Lastly, we considered only prenatal exposures. Cumulative exposures to nutrients and air pollutants from the prenatal period through early adolescence are likely important, but were beyond the scope of this analysis.

Our study also had several strengths. Use of the BKMR methodology to model our exposures of interest enabled us to detect interactions between exposure components, as well as possible non-linear dose response effects, such as those observed for Vitamin D. To our knowledge, this is the first study to examine interactions of prenatal exposures on allergic disease outcomes in this way. Our findings show that relationships between prenatal exposures and allergic disease may extend beyond early childhood and into adolescence. For many of the nutrients examined, we were able to study the effect of the nutrients from foods alone, as well as the effects of nutrients from foods and supplements combined. Direct measurement of these disease biomarkers is advantageous, as they may better capture underlying disease processes as compared to parental report of diagnoses and symptoms. Prenatal exposure mixture studies like the one presented here may ultimately be extended to incorporate effect modification by polymorphisms in relevant genes (i.e. MTHFR for folate and VDR for Vitamin D response) and/or mediation of exposure effects through intermediary “–omics” biomarker profiles (such as epigenetic, gene expression or metabolomics assessments).

In summary, we found that greater maternal intakes of vitamin D, folate, and n-3 fatty acids were associated with less airway inflammation in adolescence, while other nutrients (vitamin E), and air pollutants were associated with higher inflammation. Prenatal exposure interactions may play an important role, as evidenced by the potential synergy between vitamin E and PM_2.5_. We also observed potential protective effects of prenatal nutrients (n-3 PUFAs) and adverse associations with prenatal air pollution exposure for a second allergic disease biomarker (total IgE) in adolescence. Whenever possible, exposure interactions in prenatal life should be examined in studies of chronic disease outcomes in childhood. Understanding how prenatal exposures, particularly in the context of mixtures, influence allergic disease in childhood may ultimately inform the design of prenatal intervention trials to reduce the incidence of these disorders.

## Methods

This study involved women and their children enrolled in Project Viva, an ongoing longitudinal pre-birth cohort study. The design of this cohort study has been described in detail elsewhere^39^. The study was approved by the Brigham and Women’s Hospital Institutional Review Board and the Harvard Pilgrim Health Care Institute Institutional Review Board. All methods were performed in accordance with the guidelines and regulations of these institutional review boards. Mothers provided informed consent at enrollment and for their child during follow-up visits, and children gave verbal assent. Participants were recruited into Project Viva at 8 offices of Atrius Harvard Vanguard Medical Associates in Eastern Massachusetts between 1999 and 2002. Exclusion criteria were multiple gestation, inability to answer questions in English, plans to move out of the study area before delivery of the infant, and gestational age ≥ 22 weeks at the time of presentation for prenatal care. We saw mothers during the first and second trimesters of pregnancy, and both mothers and children at delivery and periodic postnatal research visits, most recently when children reached adolescence (the “Early Teen” visit at median 12.9 years, range 11.9 to 16.6 years). At this visit, we measured respiratory function and collected blood.

Of the 2,128 women who delivered a live infant, we excluded 270 who were missing all prenatal exposure data (nutrient intakes and air pollution exposure estimates), and 22 who were missing relevant covariates. Of the 1,836 mother-infant pairs with available exposures and covariate measures, 857 (47%) had child’s FeNO and 590 (32%) had child’s total IgE measured at the Early Teen visit. Comparison of the 2,128 participants to the 1,836 mother-infant pairs with measured exposures and covariates showed some differences. For example, maternal pre-pregnancy body mass index (BMI) was lower for participants with exposure measures (mean 24.6 vs. 26.5 kg/m^2^, t-test p value = 0.0001). A larger percentage of participants with prenatal exposures measures were white (67 vs. 39%), and highly educated (68% with a college degree vs 36%), but the two groups did not differ on maternal history of hay fever. Comparisons between the 1,836 participants with exposure and covariate data and those with FeNO and total IgE outcome data in adolescence are shown in Supplemental Table 1.

### Prenatal Dietary Nutrients and Supplement Intakes

During both the first and second trimesters, data on prenatal nutrient intakes were gathered using semi-quantitative food frequency questionnaires (FFQs) validated specifically for use in pregnancy^40^.We used the average of 1^st^ and 2^nd^ trimester FFQ derived nutrient intakes for our analyses. Use of these questionnaires in the Project Viva cohort have been described previously^41,42^. For first trimester data, the time referent was “during this pregnancy” (i.e. from the date of the last menstrual period until the assessment at a median of 9.9 weeks gestation at enrollment). The time referent for second trimester intakes collected by FFQ at the second visit (at 26–28 weeks gestation) was “during the past 3 months”. We did not administer a full FFQ that covered diet during late pregnancy, but we have found that diet was fairly stable across the first and second trimesters^43^. In addition to the FFQ, we also gathered data on vitamin and supplement intakes, including information on dose, duration of use, brand or type of multivitamin, prescribed prenatal vitamins and supplement use by the mother during both the first and second trimesters. We estimated individual nutrient intakes using the Harvard nutrient-composition database, which contains food composition values from the US Department of Agriculture, supplemented by other data sources^44^. We used mean nutrient intakes from the first and second trimesters as the exposures in the analyses, adjusted for total energy intake by the nutrient residual method^45^. If a participant completed only one FFQ, we used that value for the exposure variable.

### Prenatal Air Pollutant Exposures

We estimated third trimester air pollutant exposures of interest, residence-specific 3^rd^ trimester black carbon (BC) and PM_2.5_, using validated spatio-temporal regression models. Methodology for generating daily estimates of these exposures has been published elsewhere^46,47^. Briefly, daily residence-specific BC estimates were generated from a spatiotemporal regression model that contained land-use predictors (e.g., cumulative traffic-density within 100 m of a given location), meterological terms, and a smooth term of longitude and latitude. This model was applied separately for predictions in the warm (May-October) and cold (November-April) seasons. For BC estimates obtained using this spatiotemporal land-use regression model, the mean “out-of-sample” R^2^ was 0.73. For PM_2.5_ exposure estimates, we used a spatiotemporal prediction model that uses 10 × 10 km resolution daily satellite remote sensing data on aerosol optical depth (AOD) from the NASA MODIS satellite, land use terms, and meterological factors. The models first calibrate the remote sensing AOD data to observed PM_2.5_ ground monitoring data while accounting for land use and meterological factors. A second stage uses generalized additive models to fill in missing AOD data, due to cloud cover, snow, or other factors, using regional measured PM_2.5_, AOD values in neighboring cells, and land use terms. A final third stage uses fine-scale spatial information at 100 m resolution to account for very local traffic particle emissions. Daily PM_2.5_ exposure estimates at each residence yielded mean “out-of-sample” R^2^ of 0.83 for days having AOD remote sensing data and 0.81 for days without^48^. Participants reported their residential address at enrollment and updated it at the end of the second trimester and shortly after birth. All air pollution exposure estimates account for any moves during pregnancy. Third trimester exposure estimates were calculated by averaging daily exposures from the 188^th^ day after LMP (last menstrual period) to the day before birth. We chose to model 3^rd^ trimester air pollutant exposures, as 1) the third trimester is a very active period of lung development^49^ and therefore a biologically plausible window of vulnerability for airway inflammatory outcomes in childhood (i.e. FeNO) and 2) satellite model data for PM2.5 was available starting in the year 2000 (and therefore the 3^rd^ trimester exposure data were most complete for mothers who enrolled at the beginning of the study, which began in 1999).

### Fractional Exhaled Nitric Oxide (FeNO) Measurement

Nitric oxide (NO) is a mediator involved in chronic inflammatory diseases and Th2-mediated immune responses^50^. Measurement NO in exhaled air, as fractional exhaled nitric oxide (FeNO), is a non-invasive biomarker of airway inflammation that correlates with airway eosinophilia^51^. We measured FeNO levels with a portable electrochemical device (NIOX MINO; Aerocrine AB, Stockholm, Sweden), which is in agreement with published procedures for FeNO measurement^52^. Before FeNO measurement, participants breathed in through a nitric oxide scrubbing filter and exhaled out into the room air twice before inhaling a third time through the filter and exhaling into the FeNO analyzer. Exhaled FeNO measurements were conducted without a nose clip at a flow rate of 50 mL/s. (The last 3 seconds of the exhalation was used for FeNO measurement to ensure quantification of lower rather than upper airway FeNO). In accordance with American Thoracic Society guidelines, we did not use nose clips because blocking the nasal passages can cause exhaled nitric oxide to accumulate in the nose and potentially leak into the exhaled air stream through the posterior nasopharynx^52^. This procedure was performed twice, so that each subject had two FeNO measurements. The mean of these two measurements was used in data analysis.

### Total IgE Measurement

Of the 1,836 children with prenatal exposure assessment for air pollutants and nutrients, 706 had blood drawn at school age for additional studies, of whom 590 had sufficient sample to measure serum total IgE by using ImmunoCAP (Phadia, Uppsala, Sweden).

### Covariates

At enrollment mothers completed questionnaires reporting their education, history of atopy, smoking habits, and pre-pregnancy height and weight, from which we calculated BMI. On postpartum questionnaires, mothers reported child race/ethnicity. We obtained child sex and date of birth from hospital birth records.

### Statistical Analysis

We applied Bayesian Kernel Machine Regression (BKMR)^20^, which estimates the multivariable exposure-response function of the prenatal nutrients and air pollutant exposures with each of our two outcomes (FeNO and IgE levels). BKMR is a flexible modeling approach that allows for a non-linear and non-additive (e.g., interactive) exposure-response relationships, and which simultaneously provides measures of variable importance (posterior inclusion probabilities). Using BKMR, it is possible to estimate a flexible exposure-response function (denoted *h(z)*). For each BKMR model, we performed 10,000 iterations. We applied the hierarchical variable approach in order to rank the importance of exposure groups in association with FeNO and total serum IgE level measured at the early teen visit. Prenatal maternal exposures of interest were residence-specific air pollutants (3^rd^ trimester BC or PM_2.5_,) and nutrient intakes (1^st^ and 2^nd^ trimester averaged energy-adjusted dietary intakes of vitamins D, C, and E, β-carotene, folate, choline, and n-3 and n-6 polyunsaturated fatty acids). We conducted secondary analyses with nutrient intakes derived from supplements and food combined. We modelled all nutrients in the same exposure group except vitamin E and β-Carotene, which demonstrated associations with FeNO in the direction opposite to that observed for other nutrients. In our BKMR model specification, we grouped the exposures into 3 different groups, vitamin E and β-Carotene (group 1), all of the other nutrients (group 2), and the air pollutant (either PM_2.5_ or BC) exposure (group 3) and conducted hierarchical variable selection on these groups of exposures. In particular, under the hierarchical variable selection formulation, BKMR simultaneously identifies (1) whether there are particular groups of exposures that are associated with the outcome, and (2) which exposures within the group may be driving the association between that group of the pollutants with the outcome by calculating group-specific importance measures, which are referred to as ‘posterior inclusion probabilities’. We used posterior inclusion probabilities from the BKMR model, which is an importance score, to rank the strength of association between each exposure group and the outcome. Models were adjusted for maternal education, hay fever, pre-pregnancy BMI, and smoking in pregnancy, and child sex, race/ethnicity, season of birth, and sine and cosine of time of outcome measure to adjust for confounding by seasonal trends in both the outcome and the exposure. Exposure covariates were expressed as z-scores of log-transformed exposures and we report effects in % change in FeNO or IgE z-per interquartile range (25^th^ to 75^th^ percentile) increase in exposure. Since BC is a component of PM_2.5_, we modeled the two air pollutant exposures separately. In all, four combinations of variable types were run in BKMR analyses: Model 1: Nutrients (foods only) + BC air pollutant; Model 2. Nutrients (foods only) + PM_2.5_ air pollution; Model 3: Nutrients (foods + supplements) + BC, Model 4: Nutrients (foods + supplements) + PM_2.5_ air pollution. Models 1 and 4 are presented in the main text for the outcomes FeNO and serum total IgE. Results of models 2 and 3 are shown in the supplement. We confirmed the main effects and interactions estimated in the BKMR analyses with standard parametric regression models with interaction terms. We refer to associations as statistically significant if 95% posterior credible intervals exclude the null.

## Electronic supplementary material

Supplementary Tables
