# Supplementary material for: Relation of Prenatal Air Pollutant and Nutritional Exposures with Biomarkers of Allergic Disease in Adolescence
Source: Sci Rep. 2018 Jul 12;8:10578. doi: 10.1038/s41598-018-28216-0 (PMC6043562; doi:10.1038/s41598-018-28216-0)
Supplement: Supplementary file 1 — Supplementary Tables [file 41598_2018_28216_MOESM1_ESM.docx]

**SUPPLEMENTARY FILE**

**Relation of Prenatal Air Pollutant and Nutritional Exposures with Biomarkers of Allergic Disease in Adolescence**

Joanne E. Sordillo,^1^ Karen M. Switkowski, ^1^ Brent A. Coull,^2^ Joel Schwartz, ^2^ Itai Kloog, ^2^ Heike Gibson, ^3^ Augusto A. Litonjua,^4^ Jennifer Bobb,^5^ Petros Koutrakis, ^3^ Sheryl L. Rifas-Shiman,^1^ Emily Oken,^1^ Diane R. Gold^3,6^

^1^Division of Chronic Disease Research Across the Lifecourse (CoRAL), Department of Population Medicine, Harvard Medical School and Harvard Pilgrim Health Care Institute, Boston, MA, USA.

^2^Department of Biostatistics, Harvard T.H. Chan School of Public Health, Boston, Massachusetts, USA.

^3^Department of Environmental Health, Harvard T.H. Chan School of Public Health, Boston, Massachusetts, USA

^4^Division of Pediatric Pulmonary Medicine, University of Rochester Medical Center, Rochester, NY, USA

^5^Kaiser Permanente Washington Health Research Institute

^6^Channing Division of Network Medicine, Brigham and Women's Hospital, Harvard Medical School, Boston, Massachusetts, USA.

**Corresponding Author:**

Diane R. Gold
Email: [redrg@channing.harvard.edu](mailto:redrg@channing.harvard.edu)
Address: 181 Longwood Ave, Boston MA, 02115
Phone: (617) 525-2738

|  | Participants with Prenatal Exposure Assessment (Nutrient Intakes, Air Pollutants)  Total N=1836 | Participants with FeNO in adolescence  (age 11.9-16.6)  N=857 | Participants with  Total Serum IgE in adolescence  (age 11.9-16.6)  N=590 |
| --- | --- | --- | --- |
| Maternal Pre-pregnancy BMI (kg/m^2^), mean (SD) | 24.6 (5.3) | 24.7 (5.1) | 24.7 (5.1) |
| Maternal Education, College Degree, n (%) | 1256 (68%) | 622 (73%)* | 429 (73%)* |
| Smoking in pregnancy,  n (%) | 211 (11%) | 78 (9%)* | 59 (10%) |
| Maternal Hayfever, n (%) | 530 (29%) | 256 (30%) | 182 (31%) |
| Child’s Race/Ethnicity,  n (%) |  |  |  |
| Black | 256 (14%) | 123(15%) | 88 (15%) |
| White | 1234 (67%) | 565 (66%) | 380 (64%) |
| Hispanic | 93 (5%) | 35 (4%) | 23 (4%) |
| Other | 253 (14%) | 134 (16%) | 99 (17%) |
| Child’s sex female, n (%) | 904 (49%) | 429 (50%) | 281 (48%) |
| Season of Birth, n (%) |  |  |  |
| Winter | 433 (24%) | 203 (24%) | 143 (24%) |
| Spring | 497 (27%) | 218 (25%) | 157 (26%) |
| Summer | 503 (27%) | 249 (29%) | 167 (28%) |
| Fall | 403 (22%) | 187 (22%) | 123 (22%) |

**Supplemental Table 1. Characteristics of Mother-Infant Pairs Participating in Project Viva**

*p<0.05 for comparison with participants with prenatal exposure assessment

**Supplemental Table 2.** Posterior Inclusion Probabilities (PIPs) of Hierarchical Exposure Group for All FeNO Outcome Models

| **Hierarchical Exposure Groups*** | **Model 1**  Nutrients  (foods only) +  Black carbon | **Model 2**  Nutrients  (foods only) + PM_2.5_ | **Model 3** Nutrients (foods + supplements) + Black carbon | **Model 4** Nutrients  (foods + supplements)  + PM_2.5_ |
| --- | --- | --- | --- | --- |
| **Exposure Group 1:**  (Beta-Carotene, Vitamin E) | 0.57 | 0.34 | 0.57 | 0.56 |
| **Exposure Group 2:**  (n-3 PUFAs, n-6 PUFAs, choline, Vitamin C, Vitamin D, Folate) | 0.87 | 0.76 | 0.91 | 0.84 |
| **Exposure Group 3:**  (Air Pollutant) | 0.85 | 0.54 | 0.76 | 0.61 |

*Sources of nutrients (foods only or foods + supplements) and type of air pollutant (PM2.5 or black carbon) depend on the specific model listed in the column header.

**Supplemental Table 3.** Posterior Inclusion Probabilities (PIPs) of Hierarchical Exposure Groups for All Total Serum IgE Outcome Models

| **Hierarchical Exposure Groups*** | **Model 1**  Nutrients  (foods only) +  Black carbon | **Model 2**  Nutrients  (foods only) + PM_2.5_ | **Model 3** Nutrients  (foods + supplements) + Black carbon | **Model 4** Nutrients  (foods + supplements)  + PM_2.5_ |
| --- | --- | --- | --- | --- |
| **Exposure Group 1:**  (Beta-Carotene, Vitamin E) | 0.42 | 0.41 | 0.51 | 0.55 |
| **Exposure Group 2:**  (n-3 PUFAs, n-6 PUFAs, choline, Vitamin C, Vitamin D, Folate) | 0.46 | 0.41 | 0.31 | 0.41 |
| **Exposure Group 3:**  (Air Pollutant) | 0.75 | 0.96 | 0.70 | 0.97 |

*Sources of nutrients (foods only or foods + supplements) and type of air pollutant (PM2.5 or black carbon) depend on the specific model listed in the column header.

**Supplemental Table 4. Associations of Prenatal Nutrient Intakes (Foods only) and Prenatal Air Pollutant (3^rd^ Trimester PM_2.5_) Exposure with Allergic Disease Outcomes (FENO and Total Serum IgE) in adolescence.**

| **Hierarchical Exposure Group** | **Prenatal Exposure** (Nutrient Intakes from Foods Only and Air Pollution) | **FeNO outcome  (% change per interquartile range increase in exposure)** | | **Total Serum IgE (% change per interquartile range increase in exposure)** | |
| --- | --- | --- | --- | --- | --- |
|  |  | *Est.* | *95% CI* | *Est.* | *95% CI* |
| 1 | Vitamin E | -0.28% | -6.85 to 6.74% | -1.16% | -8.98%to 7.33% |
|  | Beta-carotene | 6.34% | -4.29 to 18.15% | 4.52% | -7.24 to 17.76% |
| 2 | Vitamin C | 3.51% | -4.60 to 12.32% | 2.65% | -6.43% to 12.62% |
|  | Folate | -7.58% | -14.64 to 0.07% | -1.50% | -10.02% to 7.82% |
|  | Choline | -0.11% | -7.04 to 7.33% | 6.55% | -4.06% to 18.33% |
|  | n-6 PUFAs | -2.72% | -13.67 to 9.62% | 5.98% | -6.11% to 19.63% |
|  | Vitamin D | -10.03% | -19.18 to 0.16% | -4.16% | -13.91% to 6.70% |
|  | n-3 PUFAs | -8.69% | -19.85 to 4.02% | -7.97% | -18.59% to 4.02% |
| 3 | PM 2.5 | **11.81%** | **0.13 to 24.86%** | **31.70%** | **11.07% to 56.16%** |

*Model adjusted for maternal pre-pregnancy BMI, education, hay fever, and pregnancy smoking status (never, former, smoked during pregnancy); season of birth, sine and cosine of date at outcome measurement; and child’s race/ethnicity and sex. Estimates with p < 0.05 are in bold.

**Supplemental Table 5. Associations of Prenatal Nutrient Intakes (Foods + Supplements) and Prenatal Air Pollutant (3^rd^ Trimester Black Carbon) Exposure with Allergic Disease Outcomes (FENO and Total Serum IgE) in adolescence.**

| **Hierarchical Exposure Group** | **Prenatal Exposure to Nutrients* and Air Pollutants** | **Model 1****  **FeNO  (% change per interquartile range increase in exposure)** | | **Model 2****  **Total Serum IgE (% change per interquartile range increase in exposure)** | |
| --- | --- | --- | --- | --- | --- |
|  |  | *Est.* | *95% CI* | *Est.* | *95% CI* |
| 1 | **Vitamin E** *(Foods + Supplements)* | 6.86% | -1.76% to 16.23% | 3.70% | -7.33% to 16.05% |
|  | **Beta-carotene**  *(Foods)* | 5.13% | -4.83% to 16.14% | 6.01% | -4.63% to 17.83% |
| 2 | **Vitamin C**  *(Foods + Supplements)* | 0.45% | -3.38% to 4.42% | -1.41% | -9.70% to 7.65% |
|  | **Folate**  *(Foods + Supplements)* | -4.09% | -12.86% to 5.55% | -5.03% | -15.06% to 6.19% |
|  | **Choline**  *(Foods)* | 0.04% | -3.71% to 3.93% | 2.29% | -6.45% to 11.84% |
|  | **n-3 PUFAs**  *(Foods)* | -10.25% | -19.81% to 0.45% | -9.73% | -19.66% to 1.42% |
|  | **Vitamin D**  *(Foods + Supplements)* | **-14.48%** | **-24.80% to -2.75%** | -0.35% | -10.56% to 11.04% |
|  | **n-6 PUFAs**  *(Foods)* | -2.26% | -10.98% to 7.31% | 5.87% | -5.09% to 18.10% |
| 3 | **Black Carbon** | 7.55% | -2.74% to 19.93% | **14.67%** | **1.66% to 29.33%** |

*Supplement data was not available for all nutrients **Model adjusted for maternal pre-pregnancy BMI, education, hay fever, and pregnancy smoking status (never, former, smoked during pregnancy); season of birth, sine and cosine of date at outcome measurement; and child’s race/ethnicity and sex. Estimates with p < 0.05 are in bold.
